# Effect of exogenous melatonin on antioxidant properties and fruit softening of ‘Fengtang’ plum fruit (*Prunus salicina* Lindl.) during storage at room temperature

**DOI:** 10.3389/fpls.2024.1348744

**Published:** 2024-03-06

**Authors:** Mingfei Zhang, Xinxia Yang, Chunmei Yin, Xingyu Lin, Kexin Liu, Kexin Zhang, Yujiao Su, Xu Zou, Ling Liao, Xun Wang, Siya He, Ruiyuan He, Guochao Sun, Jiaxian He, Bo Xiong, Zhihui Wang

**Affiliations:** College of Horticulture, Sichuan Agricultural University, Chengdu, China

**Keywords:** postharvest, plum fruit, softening, melatonin, cell wall polysaccharide, antioxidant

## Abstract

‘Fengtang‘ plums soften quickly and lose flavor after harvest. This study comprehensively evaluated the effect of exogenous melatonin on the fruit quality of ‘Fengtang’ plums. According to our findings, exogenous melatonin prevented plum fruit from losing water, delayed the decline in firmness, and preserved a high TSS/TA level. Additionally, exogenous melatonin also enhanced the activity of antioxidant enzymes and increased the non-enzymatic antioxidants, thereby further increasing the antioxidant capacity of plum fruit. Notably, exogenous melatonin delayed the degradation of covalent soluble pectin (CSP), cellulose, and hemicellulose, as well as the rise in water-soluble pectin (WSP) concentration and the activity of cell wall degrading enzymes. Further investigation using atomic force microscopy (AFM) revealed that the chain-like structure of ionic-soluble pectin (ISP) and the self-assembly network structures of CSP were depolymerized, and melatonin treatment retarded the depolymerization of pectin structures. Our results showed that exogenous melatonin preserved the postharvest quality of plum fruits by controlling fruit softness and antioxidant capacity during storage.

## 1 Introduction

Plum fruit is popular among consumers and has a promising market due to its delicate flavor and rich nutrients, including dietary fiber, vitamins, and polyphenols (Chen et al., 2021). ‘Fengtang’ plum is a superb new plum cultivar from China and its fruit exhibits a distinctive flavor with juicy, low-acid, and sweet taste similar to honey (Deng et al., 2022). However, the harvested fruits show a dramatic change in fruit texture. The transportation, storage, and marketing of plum fruits are constrained by their quick softening, which lowers fruit flavor and edibility (Lin et al., 2018). Additionally, plum fruits suffer from a series of physiological disorders and quality deterioration after harvesting, such as oxidative stress, flavor loss, and nutrient depletion (Martinez-Romero et al., 2019).

The breakdown of cell wall polysaccharides such as pectin, cellulose, and hemicellulose typically causes fruit to soften (Lin et al., 2018). Pectin, which can be further divided into water-soluble pectin (WSP), ionic-soluble pectin (ISP), and covalent-soluble pectin (CSP), has been found to be essential for maintaining intercellular adhesion and cell mechanical strength (Cybulska et al., 2015). The cell wall is additionally strengthened by the cellulose-hemicellulose network created through hydrogen bond cross-linking (Zhou et al., 2011). It has been reported that apricot fruit softening is primarily attributed to the reduced content of CSP and cellulose during postharvest storage (Fan et al., 2019). Furthermore, pectin molecules generally entangle to form a highly dynamic structure, which determines cell wall properties including stiffness and intercellular integrity (Cybulska et al., 2015). Modifications in pectin aggregates and pectin chains that occur throughout fruit ripening and storage have a significant effect on fruit quality, particularly fruit texture (Paniagua et al., 2014). The transformation of various cell wall polysaccharides is known to depend on several cell wall-degrading enzymes, including polygalacturonase (PG), pectate lyases (PL), and β-galactanases (β-GAL) (Brahem et al., 2017). The activities of these enzymes are closely related to fruit softening, whether during fruit ripening or postharvest storage.

Reactive oxygen species (ROS) metabolism is another important element influencing the quality of postharvest fruits. The excess accumulation of ROS can result in cell membrane peroxidation damage and metabolic disorders (Aghdam et al., 2019; Chotikakham et al., 2020). The balance between ROS generation and clearance is disrupted by the decreased capacity of ROS scavenging in fruit, leading to an excessive buildup of ROS. Meanwhile, the accumulation of ROS during postharvest accelerates fruit softening, senescence, and browning, thereby worsening fruit quality and hastening the senescence process (Adiletta et al., 2021). Multiple studies have demonstrated that the boost in ROS scavenging ability induced by postharvest exogenous treatments effectively delays fruit deterioration and postharvest senescence (Xie et al., 2022). Exogenous melatonin treatment increases the activities of super-oxide dismutase (SOD), peroxidase (POD), and catalase (CAT), as well as the buildup of ascorbate and total phenol, thus maintaining the fruit quality of ‘Newhall’ navel orange during postharvest (Ma et al., 2021). Additionally, hydrogen sulfide treatment could regulate the antioxidant metabolism in tomato fruits, thus maintaining good quality and delaying fruit softening (Zhong et al., 2021). Therefore, limiting fruit softening and ROS accumulation simultaneously will be a useful method for postharvest preservation.

Melatonin, an activator of the antioxidant system, has been determined to play important roles in various plant processes, such as abiotic stress response and fruit ripening (Kong et al., 2020). As an endogenous hormone found throughout the body, melatonin affects circadian rhythms, the immune system, and cancer (Guan et al., 2022). Additionally, growing research indicates that melatonin contributes significantly to improving postharvest quality (Feng et al., 2022; Saud et al., 2023). By altering the metabolism of the cell wall, melatonin administration encourages the accumulation of soluble sugars and amino acids and delays fruit softening in kiwifruit (Cao et al., 2022). However, there is still a scarcity of data on the effect of melatonin on postharvest softening and fruit quality of plums (Yan et al., 2022).

To further explore the effect of exogenous melatonin on storage quality of ‘Fengtang’ plum (*Prunus salicina* Lindl.) during storage at room temperature, this study conducted comprehensive analyses of fruit quality, antioxidant potential, cell wall polysaccharides, cell wall-degrading enzyme activity, and pectin fraction nanostructure. Moreover, the correlation between different indicators was analyzed. Our findings will provide more information for preventing fruit softening and maintaining the quality of plums.

## 2 Materials and methods

### 2.1 Plant materials and melatonin treatments

‘Fengtang’ plum fruits with uniform size, color, and ripeness as well as no physical damage were harvested in July from a well-managed orchard in Suining, Sichuan Province, China (altitude 450 m; latitude 31°10′ N; longitude 105°3′ E) and transported back to the laboratory within 4 h after harvesting. These fruits were randomly distributed into three groups (180 fruits per group). One group was dipped in distilled water for 2 min as the control group, and the other two groups were dipped in 100 μM and 200 μM melatonin solution for 2 min as melatonin-treatment groups, respectively. After dipping, all fruits were air-dried and then stored at 20-25°C with 85-90% relative humidity (simulated ambient temperature conditions). During the storage, 30 fruits in each group at 0, 3, 6, and 9 d after treatment (DAT) were randomly selected for further analysis. Fruit samples were immediately frozen in liquid nitrogen and stored at –80°C. For each treatment, there were three biological replicates, with each consisting of 10 plum fruits.

### 2.2 Determination of fruit firmness and water loss

The firmness was determined according to the previously described method with certain adjustments (Lin et al., 2018). A texture analyzer (ENS-PRO, Beijing, China) was used to measure firmness. Three spots on the equatorial section of the fruit were chosen at random and compressed by 7 mm at a speed of 60 mm/min. During the test, the greatest force produced was noted and represented in Newtons (N).

For fruit water loss, 18 moderately sized and undamaged fruits were chosen and numbered from the melatonin and control groups, respectively. The weights of the corresponding numbered fruit during storage were recorded accurately, and the data of rotten fruits during storage were excluded. Water loss (%) = [(initial weight - weight after water loss)/initial weight] × 100%.

### 2.3 Determination of fruit quality

Total soluble solids (TSS) were determined by a digital hand-held refractometer (Atago Co. Ltd., Tokyo, Japan). Titratable acid (TA) content was determined by sodium hydroxide titration; the TSS/TA was the ratio of total soluble solids to titratable acid. Each experiment was performed in triplicate.

### 2.4 Determination of antioxidant enzyme activity

Antioxidant enzyme activity was measured using 0.5 g sample. Each experiment was performed in triplicate. Briefly, precooled phosphate buffer (pH=7.8) was added to the samples, centrifuged and the supernatant was extracted for determination. The activities of SOD and POD were determined according to the previously described method. One unit (U) of SOD activity was defined as the amount of enzyme required to inhibit 50% of the photoreduction reaction of nitro blue tetrazolium (NBT). One U of the POD activity unit was defined as the amount of enzyme that caused an absorbance change of 1 in OD_470_ per minute. SOD and POD activities were expressed as U kg^-1^ (Liao et al., 2015). CAT activity was determined by ultraviolet absorption. One U of CAT activity unit was defined as the amount of enzyme required to cause 0.1 of the absorbance in OD_240_ per minute. CAT activities were expressed as U kg^-1^ (Zhang et al., 2015).

### 2.5 Determination of non-enzymatic antioxidants

The Folin-Ciocalteu colorimetric method was used to determine the total phenolic content, and the sodium nitrite-aluminum nitrate method was used to evaluate the total flavonoid content (Sarker et al., 2020). To determine APX activity was measured with a plant ascorbate peroxidase kit (G0203F, Suzhou Grace Biotechnology Co. Ltd., Suzhou, China), the contents of reduced glutathione (GSH) and oxidized glutathione (GSSG) were measured assay kits (G0207W and G0206W, Grace Biotechnology Co., Ltd, Suzhou, China), respectively, according to the manufacturer’s instructions. The ascorbic acid content was measured using a 2,6-dichloroindophenol titration (Sanchez-Mata et al., 2012). Each experiment was performed in triplicate.

### 2.6 Determination of free proline, H_2_O_2_ and malondialdehyde content

MDA and free proline contents were measured using the methods in previous studies. MDA and free proline content were measured using 0.5 g sample (Bates et al., 1973; Bian et al., 2018). The H_2_O_2_ content was determined using an H_2_O_2_ contents test kit (No. G0112W) acquired from Suzhou Grace Biotechnology (Suzhou, China). Briefly, the samples were mixed with acetone, followed by centrifuged. The supernatant was separated for H_2_O_2_ contents assay. Each experiment was performed in triplicate.

### 2.7 Extraction of cell wall materials

The cell wall material was extracted from fruits according to the previously reported method with minor modifications (Chen et al., 2017b). Each experiment was performed in triplicate. Added 30 mL of 80% ethanol (v/v) to 1 g of dried pulp and boiled the mixture for 25 min, repeating 3 times to remove the reducing sugars. Vacuum filter and incubate the filtrate with 30 mL of 90% (v/v) dimethyl sulfoxide for 15 h to remove starch. The filtrate was vacuum-filtered again with acetone and dried at 65°C to obtain cell wall material (CWM).

### 2.8 Separation and determination of cell wall polysaccharides

The cell wall polysaccharides were fractionated using the previously described method with some modifications (Chen et al., 2017a). Each experiment was performed in triplicate. Added 5 mL of sodium acetate buffer (50 mmol/L, pH 6.5) to CWM (50 mg) and oscillated for 6 h, then centrifuged for 10 min, the supernatant designated as water-soluble pectin (WSP). Next, the residual was transferred to 5 mL sodium acetate buffer (50 mmol/L, pH 6.5) which contained 50 mmol/L EDTA. To obtain ionic-soluble pectin (ISP), the mixture was centrifuged after shaking for 6 h. For covalent-soluble pectin (CSP), the residues were oscillated for 6 h with 50 mmol/L Na_2_CO_3_. The residue was oscillated for 6 hours with 5 mL of 4 mmol/L NaOH (containing 100 mmol/L NaBH_4_) to collect hemicellulose. To obtain cellulose, 1.5 mL of 80% sulfuric acid was added to the residues, which were then left for 2 h. After that, 3 mL of distilled water was added, and the mixture was hydrolyzed for 5 h at 100°C. The carbazole colorimetric method was used to determine WSP, ISP, and CSP content, and the anthrone colorimetric method was used to determine the contents of cellulose and hemicellulose (Fan et al., 2019).

### 2.9 Measurement of activities of cell wall-degrading enzyme

Polygalacturonase (PG) and cellulase (Cx) activity were measured using 0.2 g of plum pulp sample according to the PG activity assay kit (No. G0701W) and Cx activity assay kit (No. G0533W) purchased from Suzhou Grace Biotechnology Co. Ltd (Suzhou, China). Briefly, the pulp sample was added with 95% ethanol, centrifuged and the supernatant was discarded. The extraction buffer was then added after washing the residue with 80% ethanol. Finally, the sample solution was then centrifuged to separate the supernatant, which was then used to measure the enzyme activity.

The pectate lyases (PL) and β-galactanases (β-GAL) activities were measured according to the PL activity assay kit (No. G0702W) and β-GAL activity assay kit (No. G0524W). Briefly, 0.5 g pulp sample was added with extraction buffer, then, the sample solution was centrifuged and the supernatant was collected for enzyme activity determination. Each experiment was performed in triplicate.

### 2.10 Atomic force microscopy analysis

AFM determination was performed according to the previous method (Wang et al., 2021). WSP, ISP and CSP solutions were diluted to approximately 10 mg/L, maintained at 60°C for 20 min, and then vortexed for 1 min. Then, 10 μL of the sample solution was dropped onto the surface of the newly cleaved mica sheet. The mica sheet was dried overnight at 25°C. AFM (Alpha300RA, WITEC corporation, German) was used to scan the nanostructures of the pectin fractions. More than three images were taken in each treatment.

### 2.11 Statistical analysis

Data were tested using SPSS statistics software. Significance analysis was analyzed by Duncan’s multiple comparison test and independent samples t-test (*P <*0.05). The experimental data were presented as mean ± standard error.

## 3 Results

### 3.1 Effect of melatonin-treatment on fruit firmness and water loss

**Figure 1:**
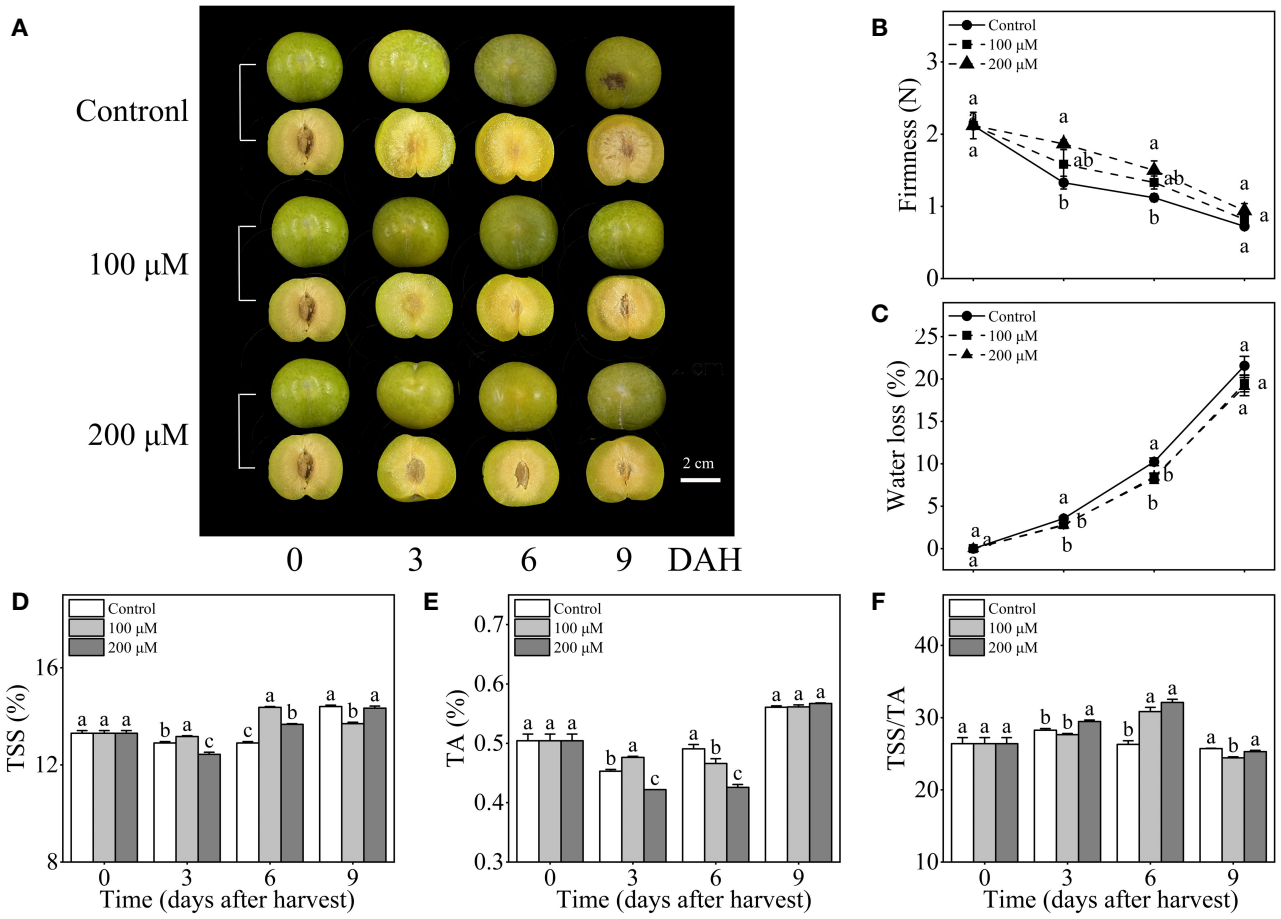
Influence of melatonin-treatment on firmness, water loss, and fruit quality of ‘Fengtang’ plum fruit. **(A)** the appearance of fruits; **(B)** firmness; **(C)** water loss; **(D)** total soluble solids (TSS); **(E)** titratable acid (TA); **(F)** TSS/TA. The vertical bars represent the SE of the means. The different letters represent the significant differences between the three groups during storage (*P <*0.05).

During storage at room temperature, the control fruits showed a waterlogging phenomenon at the periphery of the pulp (**Figure 1A**). As shown in **Figure 1B**, the firmness of three sets of ‘Fengtang’ plum fruits gradually declined throughout storage. The firmness of 200 μM melatonin-treated plum fruits was significantly higher than that of the control fruits after 3 and 6 days of harvest.

The water loss of ‘Fengtang’ plum fruits increased rapidly as the storage duration was extended. After 9 days of harvest, the control, 100 μM, and 200 μM melatonin-treated fruit experienced a maximum water loss of 21.6%, 19.4%, and 19.1%, respectively (**Figure 1C**). Additionally, melatonin treatment significantly decreased water loss in the fruit after 3 and 6 days of harvest. Consequently, the application of exogenous melatonin maintained fruit firmness and reduced water loss in ‘Fengtang’ plum fruits during short-term storage.

### 3.2 Effect of melatonin-treatment on fruit quality

The TSS and TA of ‘Fengtang’ plums displayed a tendency of decline followed by an increase, peaking at 9 d. After 3 and 6 d following harvest, the TSS content was higher in the 100 μM melatonin-treated fruits than in the control fruits. After 3 days of harvest, the TSS content in the 200 μM melatonin-treated fruits was lower compared to the control fruit, but was higher after 6 days of harvest (**Figure 1D**). In addition, during short-term storage, 200 μM melatonin-treated fruits had a lower TA content compared to the control fruit (**Figure 1E**). As a result, the TSS/TA of 200 μM melatonin-treated fruits was 22.1% higher than that of the control fruits after 6 days of harvest (**Figure 1F**).

### 3.3 Effect of melatonin-treatment on H_2_O_2_, MDA and free proline content

**Figure 2:**
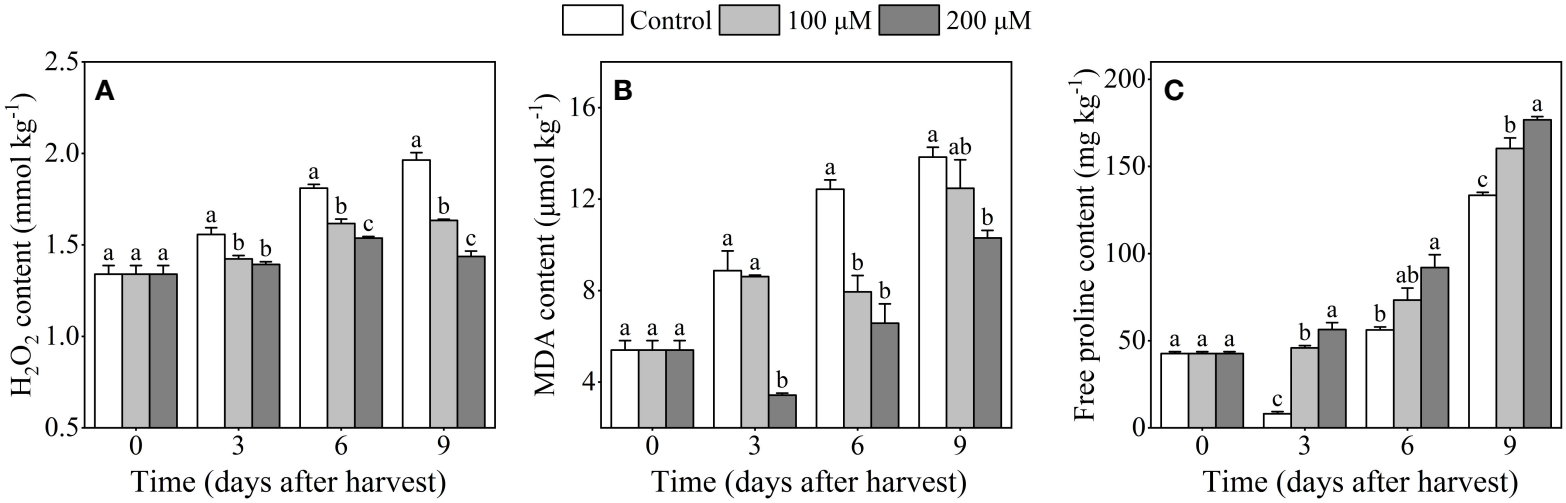
Influence of melatonin-treatment on H_2_O_2_, MDA and free proline content of ‘Fengtang’ plum fruit. **(A)** H_2_O_2_; **(B)** malondialdehyde (MDA); **(C)** free proline. The different letters represent the significant differences between the three groups during storage (*P <*0.05).

As illustrated in **Figure 2A**, the H_2_O_2_ content in the control fruits gradually increased, while melatonin treatment inhibited the increase and maintained a lower content of H_2_O_2_ than the control. At the end of storage, the H_2_O_2_ content was 16.8% and 26.8% lower in the 100 μM and 200 μM treatment groups, respectively, than that in the control group. The MDA content in control ‘Fengtang’ plums increased gradually throughout the whole storage period, and it reached its maximum after 9 days of harvest (**Figure 2B**). Compared with the control group, the MDA content in melatonin-treated fruits (especially 200 μM) increased slowly and reached 11.5 U/kg which was lower than in the other groups. The proline content in melatonin-treated fruits displayed a rising trend and was higher than that in the control fruits after 3 and 9 days of harvest (**Figure 2C**).

### 3.4 Effect of melatonin-treatment on antioxidant enzymes

**Figure 3:**
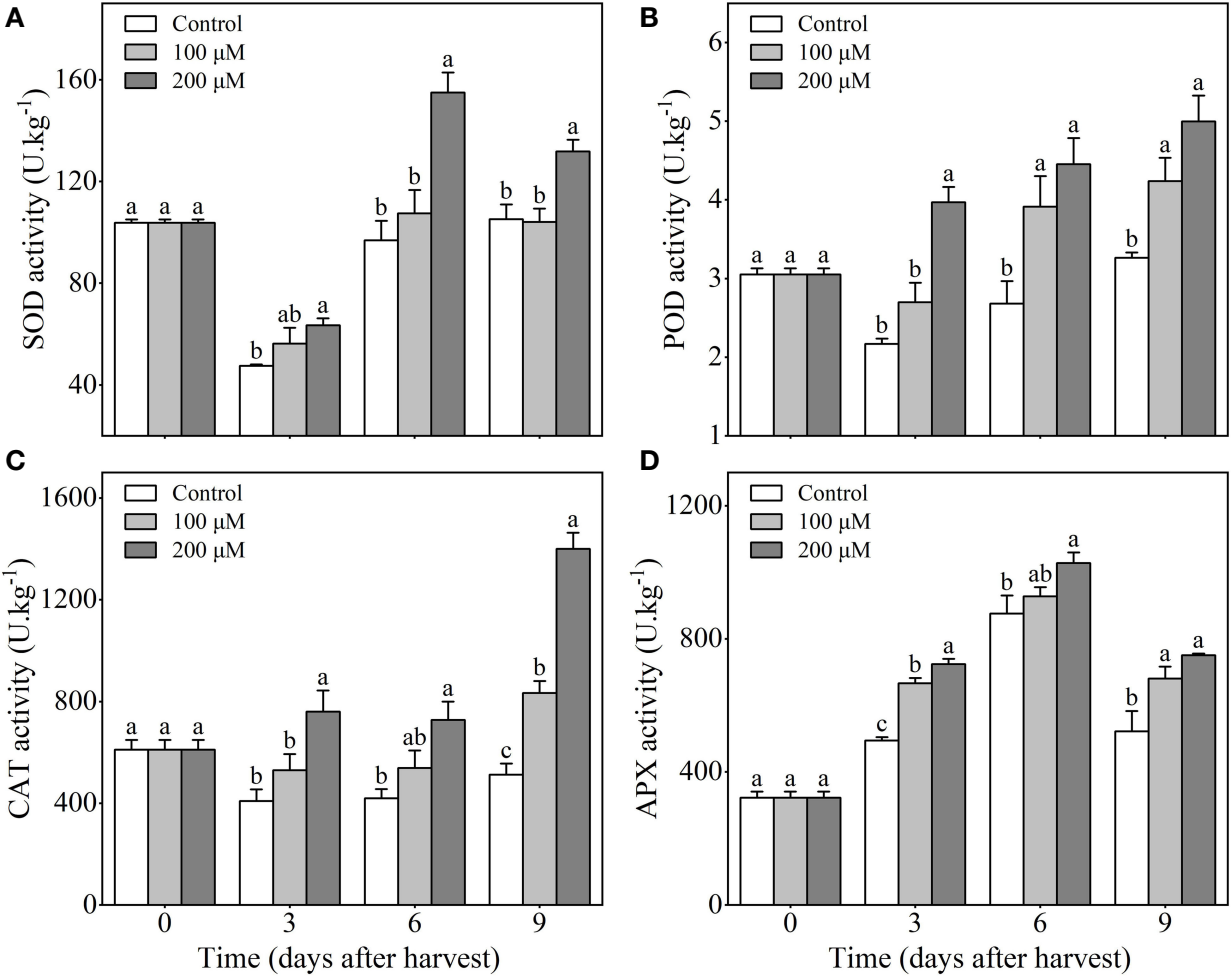
Influence of melatonin-treatment on antioxidant capacity of ‘Fengtang’ plum fruit. **(A)** superoxide dismutase (SOD); **(B)** peroxidase (POD); **(C)** catalase (CAT); **(D)** ascorbate peroxidase (APX);. The different letters represent the significant differences between the three groups during storage (P <0.05).

Fruits treated with 200 μM melatonin exhibited increased SOD activity compared to the control and 100 μM melatonin-treated fruits during storage. SOD activity decreased in three groups during the first 3 days after harvesting, but melatonin treatment prevented further decrease during the following storage period. After 9 days of harvest, the SOD activity in the 200 μM melatonin-treated fruits was 25.3% higher than that in the control fruits (**Figure 3A**). Additionally, the activity of POD in 200 μM melatonin-treated plum fruits continued to rise during storage, whereas in the control group, it decreased in the first 3 days with a subsequent upward trend. Despite that, the POD activity in 200 μM melatonin-treated fruits (4 to 5 U/kg) was still much higher than that in control fruits (less than 3.2 U/kg) throughout the storage time (**Figure 3B**). Similar to the changes in POD activity, CAT activity increased after melatonin treatment (especially 200 μM), but it remained at a stable low level in control fruits (**Figure 3C**). The APX activity of melatonin-treated fruits (especially 200 μM) was significantly higher than that of the control fruits throughout the storage period (**Figure 3D**). These findings suggested that melatonin-treatment could improve the antioxidant enzyme activity of ‘Fengtang’ plums during storage.

### 3.5 Effect of melatonin-treatment on non-enzymatic antioxidants

**Figure 4:**
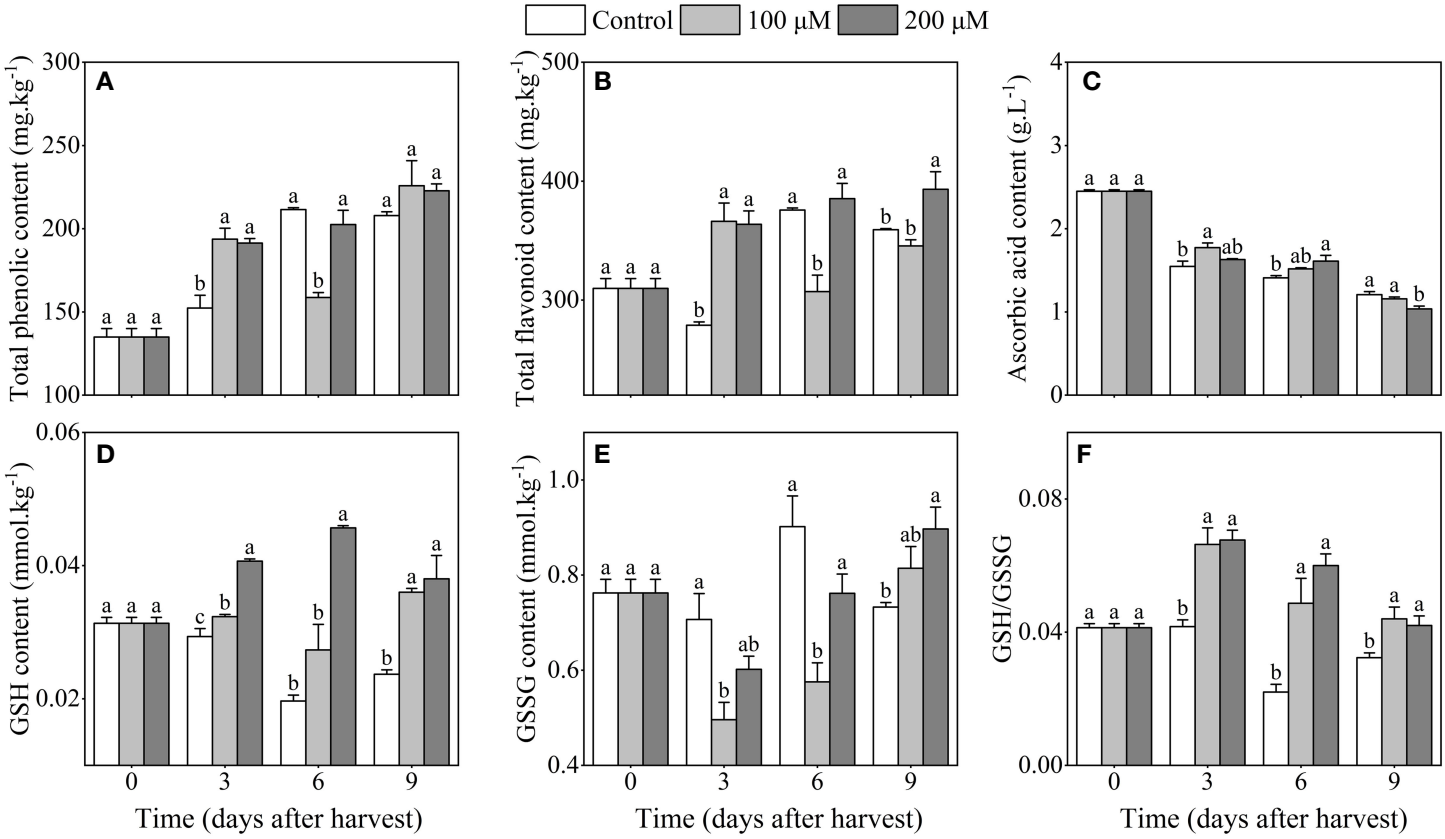
Influence of melatonin-treatment on non-enzymatic antioxidants of ‘Fengtang’ plum fruit. **(A)** total phenolics; **(B)** total flavonoids; **(C)** ascorbic acid; **(D)** reduced glutathione (GSH); **(E)** oxidized glutathione (GSSG); **(F)** GSH/GSSG. The different letters represent the significant differences between the three groups during storage (*P <*0.05).

The total phenolic content of 200 μM melatonin-treated plum fruits increased along with storage duration and was higher than the control after 3 days of harvest (**Figure 4A**). Additionally, the trend of changes in total phenolic and flavonoid content during storage was similar (**Figure 4B**). The total flavonoid content in 200 μM melatonin-treated plum fruits was considerably higher than that in control fruits after 3 and 9 days after harvest. Moreover, ascorbic acid content dropped throughout storage, and melatonin treatment delayed this decline for up to 6 days after harvest (**Figure 4C**). Melatonin-treatment promoted GSH accumulation, which was 40.0% and 76.5% higher in 200 μM melatonin-treated fruits than in the control after 3 days and 6 days of harvest, respectively (**Figure 4D**). After 9 days of storage, the content of GSSH in 200 μM melatonin-treated fruits was obviously higher than that in the control fruits (**Figure 4E**). Besides, melatonin treatment increased GSH/GSSG levels, with the GSH/GSSG levels in 200 μM melatonin-treated fruits being 1.6 and 2.7 times higher than the control after 3 days of harvest, respectively (**Figure 4F**). Thus, the application of melatonin might result in the accumulation of non-enzymatic antioxidants.

### 3.6 Effect of melatonin-treatment on contents of cell wall polysaccharides

**Figure 5:**
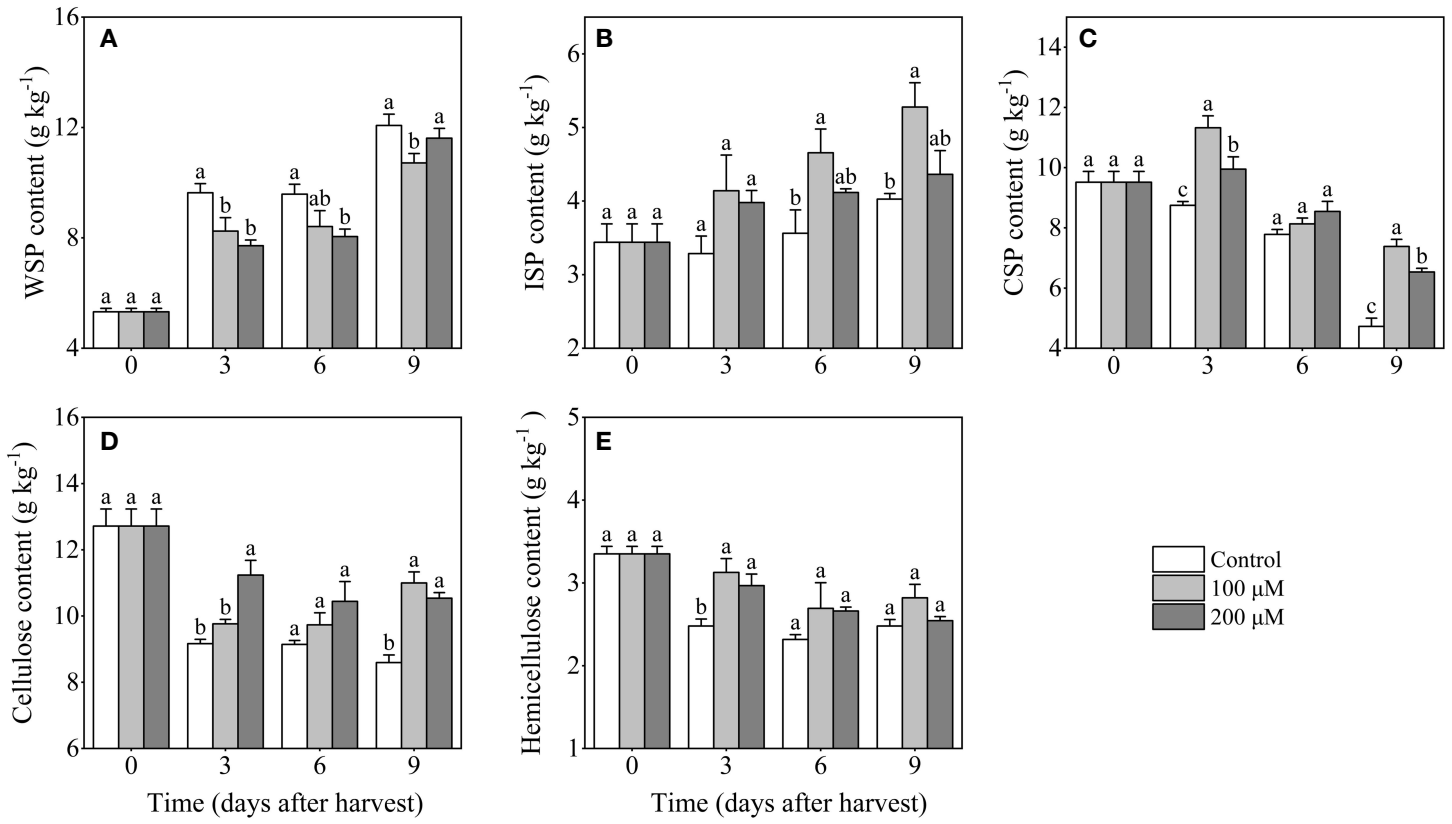
Influence of melatonin-treatment on contents of cell wall polysaccharides of ‘Fengtang’ plum fruit. **(A)** water-soluble pectin (WSP); **(B)** ionic-soluble pectin (ISP); **(C)** covalent-soluble pectin (CSP); **(D)** cellulose; **(E)** hemicellulose. The vertical bars represent the SE of the means. The different letters represent the significant differences between the three groups during storage (*P <*0.05).

The WSP content in control fruits steadily increased throughout the storage, especially in 0-3 days and 6-9 days after harvest (**Figure 5A**). After 3 and 6 days after harvest, the WSP content in 200 μM melatonin-treated fruits was 19.9% and 16.0% lower than in control fruits, respectively. The ISP content exhibited a strong increase after melatonin treatment, while it changed slightly in the control group (**Figure 5B**). In contrast, the CSP content showed an overall downward trend during storage. After melatonin treatment, the CSP content increased in the first 3 days but then decreased (**Figure 5C**). During the entire storage period, melatonin-treated fruits had a higher content of CSP than control fruits, particularly on days 3 and 9 after harvest. By the end of storage, the CSP content of melatonin-treated fruits with concentrations of 100 μM and 200 μM reached 8.8 g/kg and 6.5g/kg, respectively, which was significantly higher than that of the control group (4.5 g/kg).

The content of cellulose reduced after harvest in both control and melatonin-treated groups, whereas melatonin treatment delayed its decline. Meanwhile, melatonin treatment (200 μM) maintained a higher cellulose content after 3 and 9 days of harvest (**Figure 5D**). Notably, the content of cellulose in melatonin-treated groups was more than 11 g/kg, which is much higher than that in the control group (9.4 g/kg). Similar to the changes in cellulose content, the hemicellulose content in different groups decreased throughout storage (**Figure 5E**). In comparison with melatonin-treated fruits, a more pronounced decrease in hemicellulose content was observed in control fruits. In summary, melatonin treatment reduced the disassembly of cell wall polysaccharides (CSP, cellulose, hemicellulose) and delayed the increase of WSP in the plum fruits.

### 3.7 Effect of melatonin-treatment on cell wall-degrading enzymes activity

**Figure 6:**
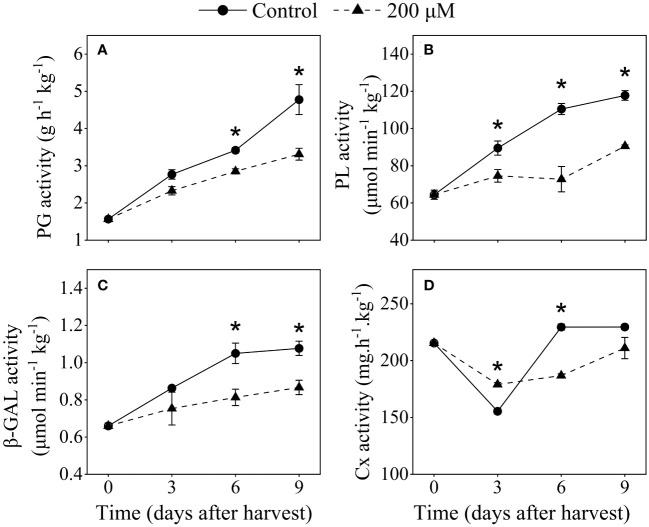
Influence of melatonin-treatment on cell wall-degrading enzymes activity of ‘Fengtang’ plum fruit. **(A)** polygalacturonase (PG); **(B)** pectate lyases (PL); **(C)** β-galactanases (β-GAL); **(D)** cellulase (Cx). The vertical bars represent the SE of the means. The asterisk * represents the significant differences between the two groups during storage (*P <*0.05).

To investigate the effect of melatonin treatment on cell wall enzymes and pectin structure in plum fruits, 200 μM melatonin-treated fruits were chosen for further analysis. The activities of PG, PL, and β-GAL significantly increased as storage progressed, and they exhibited a slower growth rate in melatonin-treated fruits compared to the control (**Figures 6A-C**). Melatonin-treated fruits showed lower PL activity than that in the control fruits. After 9 days of storage, the PL activity in the melatonin-treated group was 23% lower than that in the control group. PG and β-GAL activities were also detected in melatonin-treated fruit after 6 and 9 days postharvest. After 9 days of harvest, the PG and β-GAL activities in melatonin-treated fruit were about 20% lower than that in control fruit, respectively. Cx activity reduced in the first 3 days after harvest and subsequently increased, whereas melatonin treatment inhibited the increase in Cx activity after 6 days of harvest (**Figure 6D**).

### 3.8 Effect of melatonin-treatment on the nanostructure of WSP, ISP and CSP

**Figure 7:**
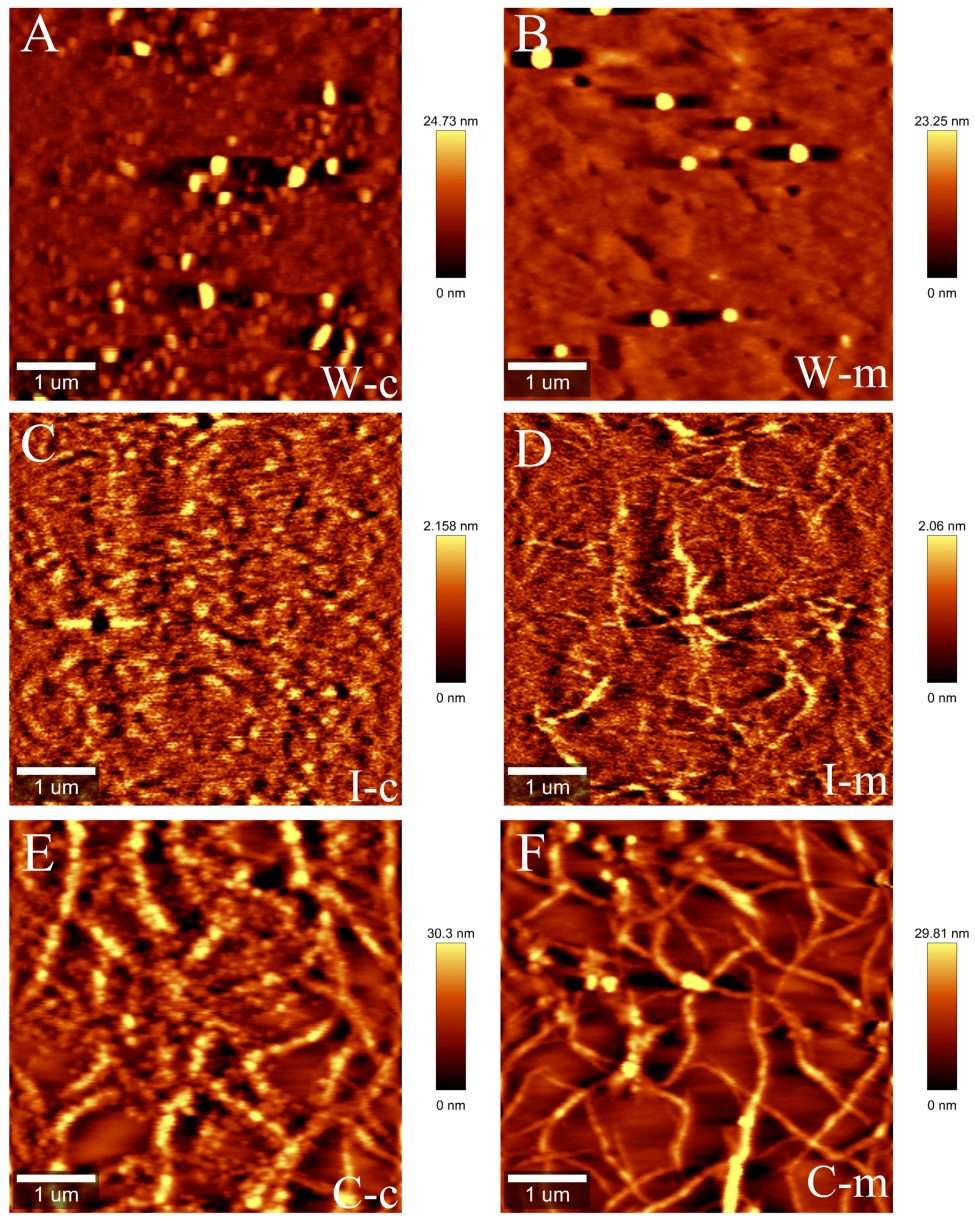
Representative atomic force microscopy (AFM) topographical images in water-soluble pectin (WSP) **(A, B)**, ionic-soluble pectin (ISP) **(C, D)** and covalent-soluble pectin (CSP) (**E, F)** of ‘Fengtang’ plum fruit after 9 days of harvest. Note: WSP (W), ISP (I), and CSP (C); c and m represent the control and the melatonin treatment, respectively.

The pectin structure determines the properties of the cell wall. We further analyzed the nanostructures of WSP, ISP and CSP after 9 days of storage. The WSP polymer on mica by AFM was found to have many small ellipses, and chains of ISP fraction were observed. In comparison to the control group, the nanostructure of IPS in the melatonin treatment group had more short chains and branches. However, the ISP aggregates in the control fruits were found to be more shortened and degraded (**Figures 7A–D**). CSP fraction formed a self-assembled network on mica that was different from the structures of WSP and ISP. The network structure of CSP in melatonin-treated fruit was interconnected and consisted of more single chains. However, the CSP fraction in the control fruit exhibited poor networking and high aggregation and had more shortened single chains and random degradation of polymers (**Figures 7E, F**).

### 3.9 Correlation analysis

**Figure 8:**
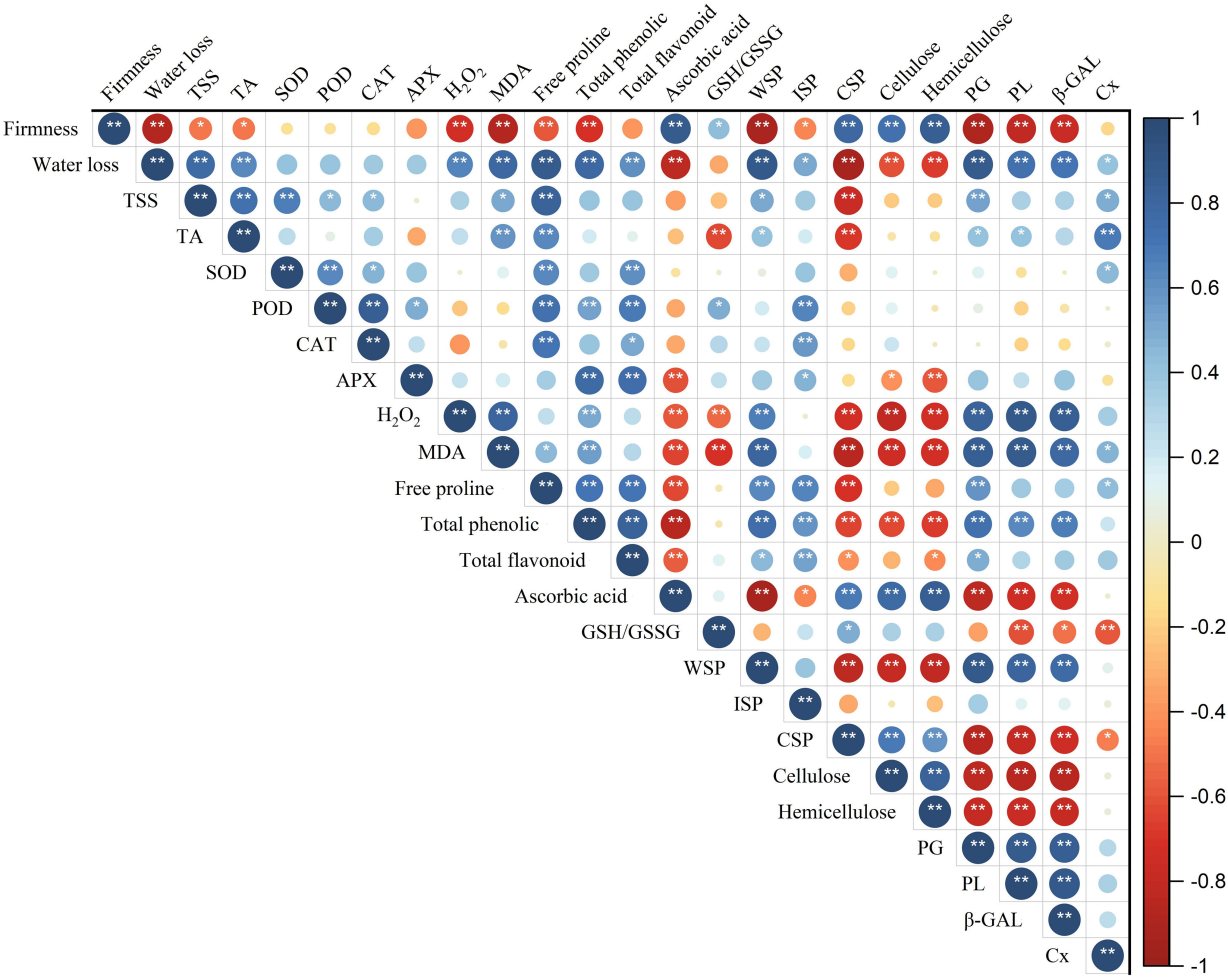
Correlation analysis of the indicators of melatonin-treated ‘Fengtang’ plums after harvest. Blue indicates a positive correlation and red indicates a negative correlation (* *P* < 0.05, ** *P* < 0.01). Total soluble solids (TSS); titratable acid (TA); superoxide dismutase (SOD); peroxidase (POD); catalase (CAT); ascorbate peroxidase (APX); reduced glutathione (GSH); oxidized glutathione (GSSG); malondialdehyde (MDA); water-soluble pectin (WSP); ionic-soluble pectin (ISP); covalent-soluble pectin (CSP); polygalacturonase (PG); pectate lyases (PL); β-galactanases (β-GAL); cellulase (Cx).

Pearson correlation coefficient analysis was further constructed to analyze the correlation between fruit quality, reactive oxygen species and cell wall metabolism during storage of ‘Fengtang’ plum fruit. As shown in **Figure 8**, the fruit firmness, as well as water loss, was significantly positively correlated with ascorbic acid, CSP, cellulose, and hemicellulose. Conversely, they were negatively correlated with water loss, MDA, total phenolic, proline, WSP, PG, PL, and β-GAL. Besides, the content of H_2_O_2_ was found to be significantly positively correlated with MDA, total phenolic, WSP, and the activity of three cell wall-degrading enzymes (PG, PL and β-GAL), but negatively correlated with CSP, cellulose, hemicellulose, ascorbic acid and GSH/GSSH.

## 4 Discussion

**Figure 9:**
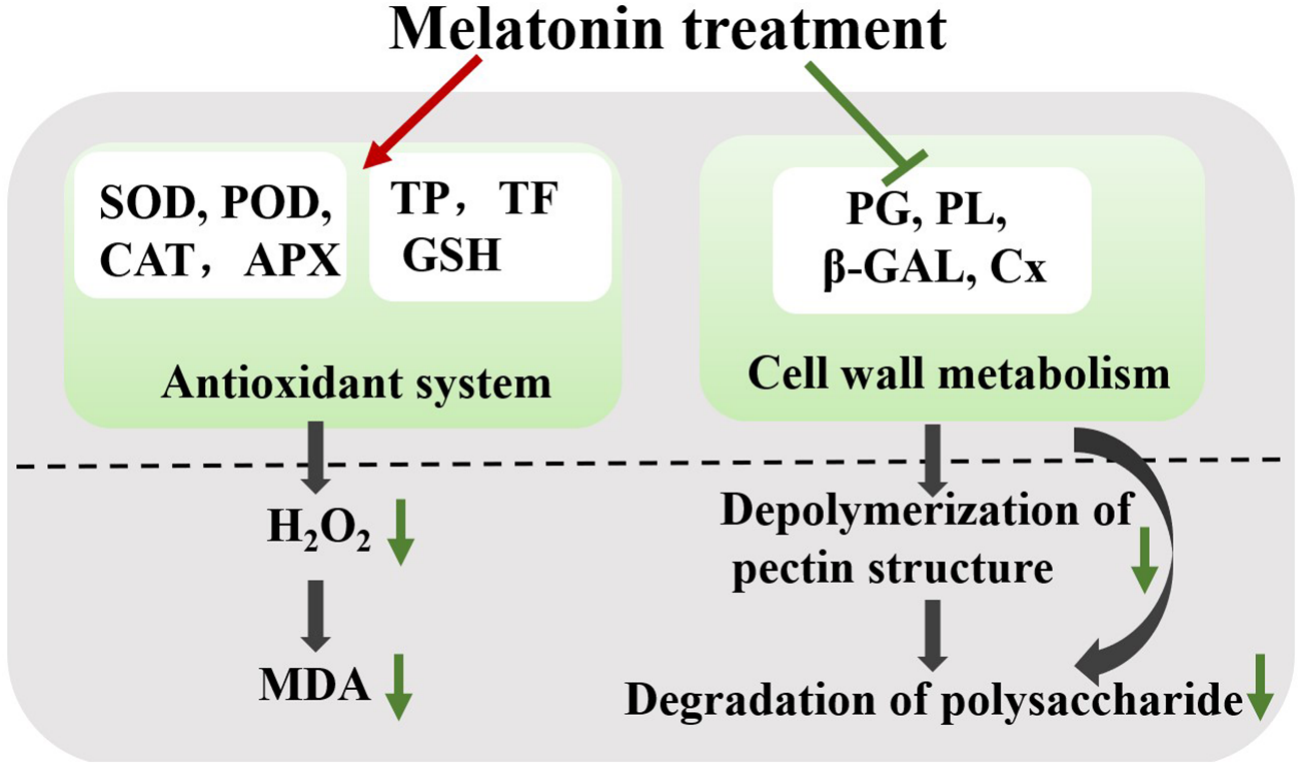
Effect of melatonin-treatment on fruit softening and antioxidant capacity in ‘Fengtang’ plums. Green lines and arrows represent inhibition; red lines represent promotion. SOD, superoxide dismutase; POD, peroxidase; CAT, catalase; TP, total phenols; TF, total flavonoids; MDA, malondialdehyde; PG, polygalacturonase; PL, pectate lyases; β-GAL, β-galactanases; Cx, cellulase.

During storage, the fruit undergoes quality deterioration, such as softening, water loss, flavor loss, and nutrient depletion (Seymour et al., 2013). Our results revealed that exogenous melatonin could be involved in the simultaneous regulation of antioxidant capacity and fruit softening, thereby effectively maintaining plum quality after harvest (**Figure 9**).

The burst of ROS (particularly H_2_O_2_) after harvest is considered to be one of the most important factors affecting fruit deterioration and accelerating postharvest senescence (Guo et al., 2020). Inhibition of ROS production is thought to be an effective strategy for preserving postharvest fruit quality (Meitha et al., 2020). In our study, we discovered that melatonin treatment suppressed the accumulation of H_2_O_2_ in plum fruits during storage duration. Meanwhile, the product of membrane lipid peroxidation, namely Malondialdehyde (MDA), also showed low content in plum fruits treated with melatonin (**Figure 2A**). Proline is an important osmoregulator for plants against abiotic stresses and plays a role in increasing dehydration tolerance and stability of cell membranes. In this study, free proline content was higher in melatonin-treated ‘Fengtang’ plum fruits than in the control (**Figure 2**). These results were similar to other studies that analyzed different fruits such as sweet cherry and apple (Wang et al., 2019; Onik et al., 2021). Herein, the determination of enzymatic and non-enzymatic antioxidant activity revealed that melatonin treatment increased the activity of antioxidant enzymes and accelerated the accumulation of non-enzymatic antioxidants (**Figures 3**, **4**). Because of its antioxidative properties, melatonin has been widely used in fruit postharvest preservation (Miranda et al., 2020). Additionally, as a safe biodegradable molecule, melatonin has no impact on food safety (Sharif et al., 2018; Gao et al., 2022). The concentration of melatonin used in this study was within the effective range. Therefore, our findings further confirm that melatonin treatment promotes the activation of the antioxidant scavenging system, thereby maintaining fruit quality during short-term postharvest.

Fruit softening and the breakdown of cell wall polysaccharides are intimately connected processes. The soluble pectin content rises, but the insoluble pectin content drops as the fruit softens (Song et al., 2016). In this study, we found that the WSP content increased, while the contents of CSP, cellulose, and hemicellulose decreased during storage (**Figure 5**). Similar results have been reported in a previous study on the postharvest storage of Younai plums (Lin et al., 2018).

Our findings indicated that melatonin treatment retarded the degradation of pectin and cellulose in plum fruits. During postharvest, the levels of ISP and CSP as well as cellulose and hemicellulose were higher in melatonin-treated fruits compared to control fruits (**Figure 5**). In general, the alterations in pectin fractions result in pectin cohesion loss and cell wall disintegration (Cybulska et al., 2015). Distinct from soluble-pectin fractions (WSP and ISP), the maintenance of CSP content is thought to be a key factor in delaying fruit softening during postharvest. CSP possesses self-assembly properties and is covalently linked to cell wall polysaccharides (Pose et al., 2012). According to our results, the CSP content in melatonin-treated fruits decreased slowly and remained higher than that in control fruits. Furthermore, after melatonin treatment, the micromorphology of pectin showed that the CSP fraction exhibited dense networking and high aggregation (**Figure 6**). Several key enzymes (such as PG, PL, and β-Gal) involved in pectin metabolism showed low activity in melatonin-treated plum fruits (**Figure 5**). The coordinated responses of these degrading enzymes jointly modulate the depolymerization and solubilization of pectin, reducing the postharvest quality of fruits such as blueberry and Jujuba (Tang et al., 2020; Qu et al., 2022). Hence, we assumed that the inhibition of enzymatic degradation induced by exogenous melatonin delayed the changes in the structure and content of pectin, which was mainly responsible for maintaining fruit firmness during storage.

The accumulation of H_2_O_2_ is commonly associated with fruit softening, such as grapevine and strawberry (Pilati et al., 2014; Zhang et al., 2022). Melatonin delays eggplant fruit softening and senescence by enhancing antioxidant capacity and inhibiting cell wall degradation (Song et al., 2022). Meanwhile, attacking the cell wall polysaccharides by ROS is one of the main reasons for accelerating western fruit softening (Fry et al., 2001). Herein, we also found that the activation of the H_2_O_2_ scavenging system showed a positive correlation with the maintenance of fruit firmness (**Figure 8**). Melatonin treatment activated antioxidant enzyme activities and promoted the accumulation of total phenols and total flavonoids, thereby inhibiting the accumulation of H_2_O_2_ and MDA. Meanwhile, many studies have revealed that melatonin treatment inhibited firmness decline and suppressed ethylene production and respiration rate in plum fruit (Yan et al., 2022). The mechanism of melatonin regulating fruit softening and other fruit quality traits still needs further research.

## 5 Conclusions

Our results indicated that exogenous melatonin maintained the postharvest quality of plum fruits by synergistically regulating fruit softening and antioxidant capacity during storage. Notably, melatonin treatment inhibited the activity of cell wall-degrading enzymes, thereby attenuating the degradation of cell wall polysaccharides (CSP, cellulose and hemicelluloses). Further nanostructure analysis revealed that melatonin treatment postponed the depolymerization of ISP and CSP fractions in the pectin structure. Meanwhile, melatonin treatment suppressed reactive oxygen species (H_2_O_2_) by increasing the activity of antioxidant enzymes (SOD, POD, CAT) and non-enzymatic antioxidants (total phenols, total flavonoids). In conclusion, exogenous melatonin maintained the freshness of plum fruits by simultaneously delaying softening and enhancing antioxidant capacity.

## Data availability statement

The raw data supporting the conclusions of this article will be made available by the authors, without undue reservation.

## Author contributions

MZ: Conceptualization, Funding acquisition, Investigation, Resources, Validation, Writing – review & editing. XY: Formal Analysis, Investigation, Writing – original draft. CY: Investigation, Writing – review & editing. XL: Investigation, Writing – review & editing. KL: Investigation, Writing – review & editing. KZ: Investigation, Writing – review & editing. YS: Investigation, Writing – review & editing. XZ: Investigation, Writing – review & editing. LL: Resources, Supervision, Writing – review & editing. XW: Resources, Supervision, Writing – review & editing. SH: Resources, Supervision, Writing – review & editing. RH: Investigation, Writing – review & editing. GS: Resources, Supervision, Writing – review & editing. JH: Resources, Supervision, Writing – review & editing. BX: Resources, Supervision, Writing – review & editing. ZW: Conceptualization, Funding acquisition, Writing – review & editing.
